# Understanding the Decline of Incident, Active Tuberculosis in People With Human Immunodeficiency Virus in Switzerland

**DOI:** 10.1093/cid/ciad330

**Published:** 2023-05-31

**Authors:** Marius Zeeb, Burcu Tepekule, Katharina Kusejko, Claudine Reiber, Marisa Kälin, Lena Bartl, Julia Notter, Hansjakob Furrer, Matthias Hoffmann, Hans H Hirsch, Alexandra Calmy, Matthias Cavassini, Niklaus D Labhardt, Enos Bernasconi, Dominique L Braun, Huldrych F Günthard, Roger D Kouyos, Johannes Nemeth, the Swiss HIV Cohort Study, Jan Meier, Yves Schäfer, Océane Follonier, Danièle Perraudin, Marianne Amstad

**Affiliations:** Division of Infectious Diseases and Hospital Epidemiology, University Hospital Zurich, Zurich, Switzerland; Institute of Medical Virology, University of Zurich, Zurich, Switzerland; Department of Ecology and Evolutionary Biology, Princeton University, Princeton, New Jersey, USA; Division of Infectious Diseases and Hospital Epidemiology, University Hospital Zurich, Zurich, Switzerland; Institute of Medical Virology, University of Zurich, Zurich, Switzerland; Division of Infectious Diseases and Hospital Epidemiology, University Hospital Zurich, Zurich, Switzerland; Division of Infectious Diseases and Hospital Epidemiology, University Hospital Zurich, Zurich, Switzerland; Division of Infectious Diseases and Hospital Epidemiology, University Hospital Zurich, Zurich, Switzerland; Division of Infectious Diseases and Hospital Epidemiology, Cantonal Hospital St Gallen, St. Gallen, Switzerland; Department of Infectious Diseases, Inselspital, Bern University Hospital, University of Bern, Bern, Switzerland; Clinic for Infectious Diseases, Cantonal Hospital Olten, Olten, Switzerland; Division of Infectious Diseases and Hospital Epidemiology, University Hospital Basel, Basel, Switzerland; Clinical Virology, Laboratory Medicine, University Hospital Basel, Basel, Switzerland; Department Biomedicine, Transplantation and Clinical Virology, University of Basel, Basel, Switzerland; HIV/AIDS Unit, Division of Infectious Diseases, University Hospital Geneva, University of Geneva, Geneva, Switzerland; Faculty of Medicine, University of Geneva, Geneva, Switzerland; Division of Infectious Diseases, University Hospital Lausanne, University of Lausanne, Lausanne, Switzerland; Division of Clinical Epidemiology, Department of Clinical Research, University Hospital Basel, Basel, Switzerland; University of Basel, Basel, Switzerland; Faculty of Medicine, University of Geneva, Geneva, Switzerland; Division of Infectious Diseases, Ente Ospedaliero Cantonale Lugano, University of Geneva and University of Southern Switzerland, Lugano, Switzerland; Division of Infectious Diseases and Hospital Epidemiology, University Hospital Zurich, Zurich, Switzerland; Institute of Medical Virology, University of Zurich, Zurich, Switzerland; Division of Infectious Diseases and Hospital Epidemiology, University Hospital Zurich, Zurich, Switzerland; Institute of Medical Virology, University of Zurich, Zurich, Switzerland; Division of Infectious Diseases and Hospital Epidemiology, University Hospital Zurich, Zurich, Switzerland; Institute of Medical Virology, University of Zurich, Zurich, Switzerland; Division of Infectious Diseases and Hospital Epidemiology, University Hospital Zurich, Zurich, Switzerland

## Abstract

**Background:**

People with human immunodeficiency virus type 1 (HIV-1) (PWH) are frequently coinfected with *Mycobacterium tuberculosis* (MTB) and at risk for progressing from asymptomatic latent TB infection (LTBI) to active tuberculosis (TB). LTBI testing and preventive treatment (TB specific prevention) are recommended, but its efficacy in low transmission settings is unclear.

**Methods:**

We included PWH enrolled from 1988 to 2022 in the Swiss HIV Cohort study (SHCS). The outcome, incident TB, was defined as TB ≥6 months after SHCS inclusion. We assessed its risk factors using a time-updated hazard regression, modeled the potential impact of modifiable factors on TB incidence, performed mediation analysis to assess underlying causes of time trends, and evaluated preventive measures.

**Results:**

In 21 528 PWH, LTBI prevalence declined from 15.1% in 2001% to 4.6% in 2021. Incident TB declined from 90.8 cases/1000 person-years in 1989 to 0.1 in 2021. A positive LTBI test showed a higher risk for incident TB (hazard ratio [HR] 9.8, 5.8–16.5) but only 10.5% of PWH with incident TB were tested positive. Preventive treatment reduced the risk in LTBI test positive PWH for active TB (relative risk reduction, 28.1%, absolute risk reduction 0.9%). On population level, the increase of CD4 T-cells and reduction of HIV viral load were the main driver of TB decrease.

**Conclusions:**

TB specific prevention is effective in selected patient groups. On a population level, control of HIV-1 remains the most important factor for incident TB reduction. Accurate identification of PWH at highest risk for TB is an unmet clinical need.

## Teaser

Incident active tuberculosis (TB) in people with human immunodeficiency virus type 1 (HIV-1) is declining in Switzerland, which is only in part explained by TB specific prevention. Controlling the HIV-1 infection and immune system restoration contribute to the TB decline.

## BACKGROUND

Human immunodeficiency virus type 1 (HIV-1) and *Mycobacterium tuberculosis* (MTB) remain a global public health problem [1, 2]. In people with HIV (PWH), MTB infection progresses faster and disseminates earlier [3–5]. Active tuberculosis (TB) is an AIDS defining disease and remains globally the most frequent cause of death in PWH [6]. The natural history of MTB infection in individuals without HIV is different: more than 90% of exposed humans contain or even clear the infection. The state of a contained infection, which also affects many PWH, is often referred to as latent TB infection (LTBI) [7]. The binary discrimination between LTBI and TB is an oversimplification but has been used for decades to gauge the risk for TB [8].

Progression from LTBI to TB is a slow process taking months to years in immunocompetent populations. The long incubation period is a window of opportunity for preventive treatment. Preventing TB has major advantages: on an individual level, preventive treatment requires shorter exposure to fewer antibiotics and prevents tissue destruction; on a public health level, prevention of TB cases reduces infectiousness and transmission [9]. However, preventive treatment also requires use of antibiotics with potential side effects and substantial risk for interactions with antiretroviral therapy (ART) [10, 11]. Therefore, accurately identifying PWH at high risk of TB remains a priority.

Over the 40 years of the HIV pandemic, 2 immune based tests for the diagnosis of LTBI were used: the over 100-year-old tuberculin skin test (TST), which was replaced by the interferon gamma release assay (IGRA) from 2007 onward in some countries and/or health care providers [12]. Most guidelines recommend testing followed by preventive therapy in PWH tested positive for LTBI [13, 14]. TB specific prevention prevents cases of incident active TB but its effectiveness varies (eg, risk ratio [RR] 0.38 in treated LTBI positive and 0.89 in treated LTBI negative compared to placebo) [15–17].

In the current study, we aimed to assess the risk factors for the development of TB in PWH in the current era of ART in a low-endemicity setting using the Swiss HIV Cohort Study (SHCS) [18, 19]. The SHCS encompasses over 21 000 PWH from the beginning of the HIV pandemic, prior to the development of ART, until today with modern single tablet and injectable regimens. It remains, to our best knowledge, unique for systematically reporting LTBI tests, preventive treatment, and long-term outcomes of MTB infection [17]. Thus, the SHCS is ideally suited to investigate the efficacy of MTB prevention strategies.

## METHODS

### Swiss HIV Cohort Study

Our population consists of PWH enrolled in the SHCS, a multicentric prospective cohort study with biannual follow-ups [19].

### Definitions

TB is defined by microbiological detection of *M. tuberculosis* in the context of clinical signs and symptoms. We divided TB into prevalent TB and incident TB. “Prevalent TB” cases are identified at SHCS enrolment (ie, typically at HIV diagnosis) and are as such not preventable. We defined TB after 6 months or later as preventable (“incident TB”). We excluded incident TB cases due to relapses or reinfections after a successful treatment. We defined LTBI as a positive IGRA or TST at least 6 months before occurrence of incident TB or censor date in PWH without TB. Preventive treatment was defined as initiation of treatment with rifampicin, rifabutin, isoniazid, rifapentine, or pyrazinamide. Our preventive treatment analysis focused primarily on its effectiveness, that is, the effect of the real-world practice. We also assessed its efficacy, that is, restricting the analysis to PWH treatment adherent for the full durations without reinfection possibility. ART adherence was used as proxy for preventive TB treatment adherence and frequent tropical traveling, between completed preventive treatment and pre-TB, as a proxy for reinfection.

**Figure 1.**
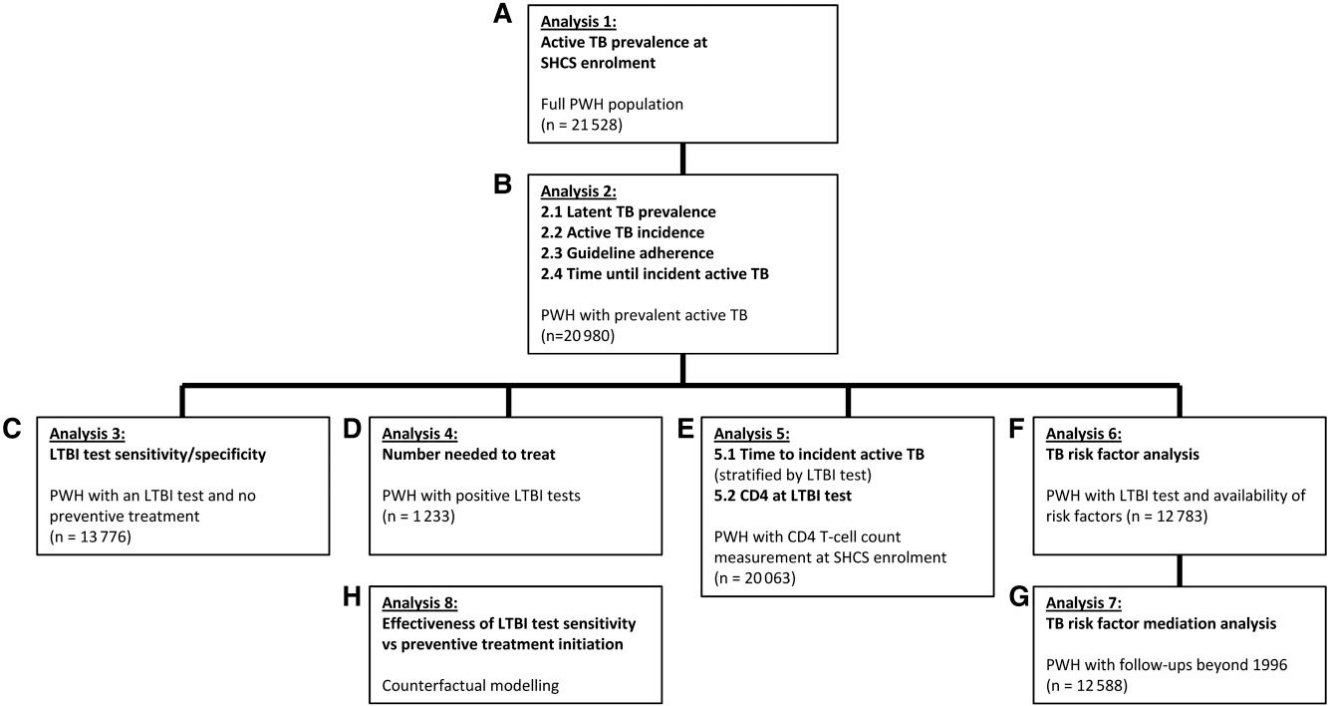
Depiction of the study design, showing workflow of the analysis, and chosen subpopulations with its patient number. Abbreviations: HIV, human immunodeficiency virus; LTBI, latent TB infection; PWH, people with HIV; SHCS, Swiss HIV Cohort study; TB, tuberculosis.

For the incident TB risk factor analysis, we included factors displayed in Table 1 as time updated covariables (analysis 6./7., Figure 1*F*, *G*): CD4^+^ T-cell count, LTBI positive versus negative test, having received/started preventive treatment, HIV viral load, body mass index, and age. As time constant we included ethnicity and HIV transmission group.

**Table 1.** Characteristics of PWH Included in the Risk Factor Analysis at Time of Their First LTBI Test

n	No Active TB	Active TB	*P* Value
	12 710	73	
Age (median [IQR])	37 [31, 45]	34 [29, 39]	.001
BMI (%)	…	…	.092
< 18.5	759 (6.0)	8 (11.0)	
18.5–25	8315 (65.4)	50 (68.5)	
> 25	3636 (28.6)	15 (20.5)	
CD4 T-cell count (median [IQR]) [cells/µl]	396 [240, 593]	321 [184, 487]	.028
RNA plasma viral load (%) [copies/ml]	…	…	.003
< 50	4129 (32.5)	9 (12.3)	
50–400	1040 (8.2)	6 (8.2)	
400–1000	530 (4.2)	4 (5.5)	
> 1000	7011 (55.2)	54 (74.0)	
Ethnicity (%)	…	…	.001
White	9747 (76.7)	40 (54.8)	
Black	1714 (13.5)	22 (30.1)	
Other	1249 (9.8)	11 (15.1)	
Most likely HIV acquisition source (%)	…	…	.002
MSM	5170 (40.7)	15 (20.5)	
Male het	2125 (16.7)	12 (16.4)	
Female het	3634 (28.6)	29 (39.7)	
Male other	1781 (14.0)	17 (23.3)	
Region of origin (%)			.001
European	10 022 (78.9)	42 (57.5)	
Sub-Saharan Africa	1454 (11.4)	21 (28.8)	
Other	1234 (9.7)	10 (13.7)	
Calendar year of first LTBI test (%)	…	…	.001
< 1996	5026 (39.5)	40 (54.8)	
1996–2007	2528 (19.9)	22 (30.1)	
> 2007	5156 (40.6)	11 (15.1)	

Abbreviations: BMI, body mass index; HIV, human immunodeficiency virus; IQR, interquartile range; LTBI, latent TB infection; MSM, men who have sex with men; PWH, people with HIV; TB, tuberculosis.

We defined “adherent” to TB specific prevention (analysis 2.3., Figure 1*B*) as a positive LTBI test with subsequent prescription of preventive treatment or a LTBI negative test with or without subsequent prescription of preventive treatment.

### TB Incidence and Prevalence

For each year between 1988 and 2022 we calculated the prevalence of TB, within 6 months after SHCS enrolment (prevalent TB) (analysis 1., Figure 1*A*) and the prevalence of LTBI among PWH within six months after SHCS enrolment (analysis 2.1., Figure 1*B*).

We calculated the incidence rate for incident TB ≥ 6 months after SHCS enrollment per 1000 person-years (analysis 2.2., Figure 1*B*) and the time from SHCS enrolment until incident TB (analysis 2.4., Figure 1*B*).

### Specificity and Sensitivity of LTBI Testing

For specificity and sensitivity calculation of LTBI testing for the prediction of active TB we restricted to PWH without preventive treatment (analysis 3., Figure 1*C*).

We compared the CD4 T-cell count between LTBI test result, that is, positive, negative, or no test at SHCS enrolment (analysis 5.2., Figure 1*E*).

We analyzed time from SHCS enrolment until incident TB stratified by positive-, negative-, or no LTBI test (analysis 5.1., Figure 1*E*).

### TB-Specific Prevention and Number Needed to Treat

We divided management of PWH into TB specific prevention (non-)adherent (analysis 2.3, Figure 1*B*), as defined above. Furthermore, we stratified by SHCS enrolment year, that is, before 1996 (before triple combination ART), 1996–2007, and after 2007 (IGRA was introduced). We then compared the incident TB cases proportion within strata using Fisher's-exact-test.

We calculated the number needed to treat (NNT) with preventive treatment in PWH with a positive LTBI test (analysis 4., Figure 1*D*) for TB free survival time of 16 years using the Kaplan-Meier approach.

### Risk Factor Analysis

We determined hazard ratios (HR) of the risk factors (analysis 6., Figure 1*F*) LTBI testing, and preventive treatment on acquiring incident TB, by univariable and multivariable time-updated cox proportional hazard models. We defined the time at risk from the date of the first available LTBI test (negative- or positive), at least 6 months before incident TB or last follow-up, until the date of incident TB or last follow-up.

We defined the binary outcome as the occurrence of incident TB. We censored PWH without incident TB until their last available follow-up date or 1 August 2022. We excluded PWH with prevalent TB, with undeterminable LTBI status, or without complete risk factor data. For partially missing data we extrapolated with last-value carried forward/backward.

### TB-Specific Prevention Versus LTBI Test Sensitivity

We established a 5-compartment model using coupled ordinary differential equations (Supplementary Figure 7) with the R package deSolve, to simulate counterfactual scenarios [20]. This allowed us to compare hypothetical preventive treatment initiation improvements to hypothetical LTBI test sensitivity improvements (analysis 8., Figure 1*H*).

### Multiple Mediation Analysis

We performed multiple mediation analysis (analysis 7., Figure 1*G*) using the R package MMA [21]. We used a simplified version of our time updated Cox proportional hazard model and included only the time after 1996 (due to ART availability).

## RESULTS

### Time Trends of LTBI Testing, Incident- and Prevalent TB

**Figure 2.**
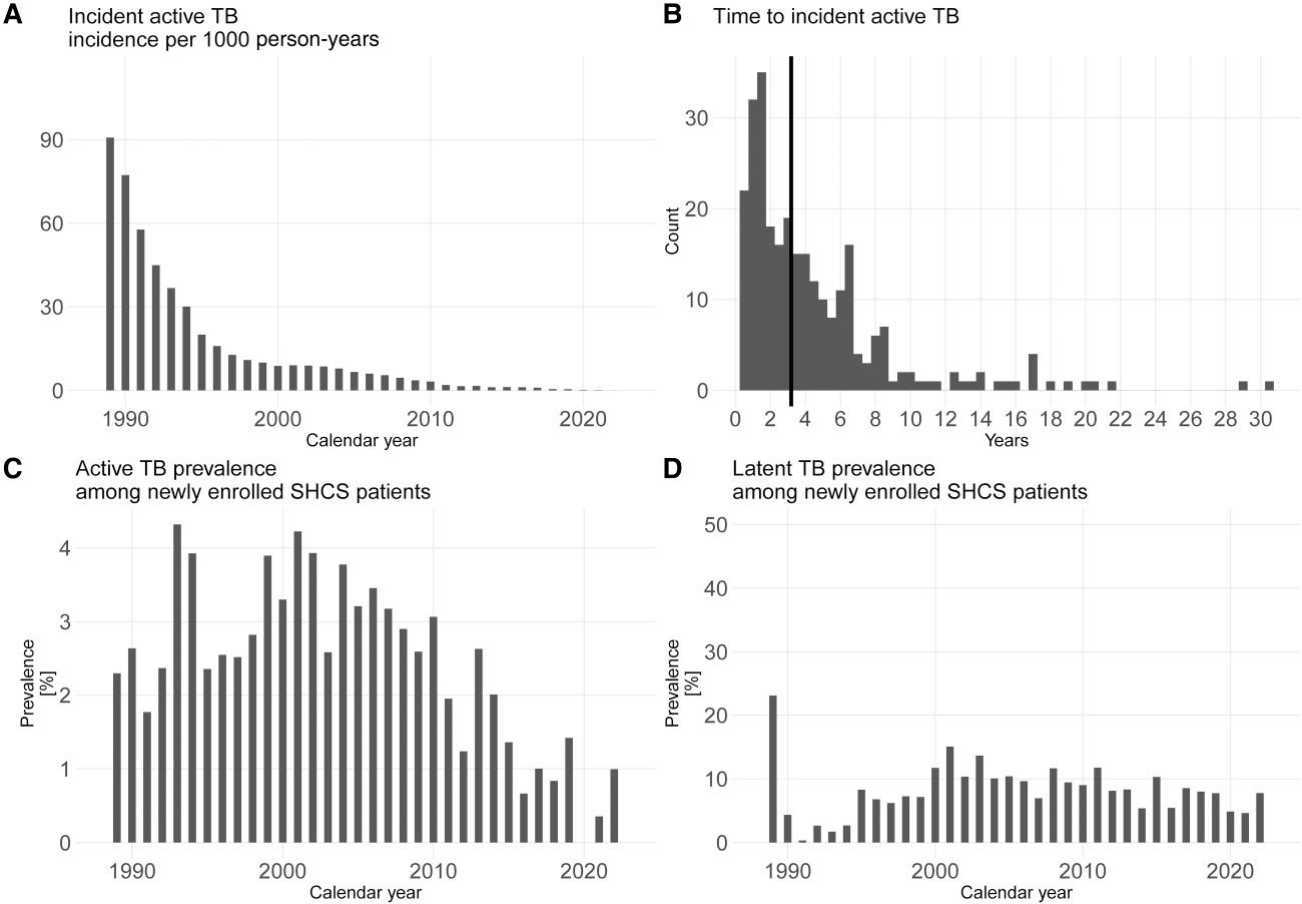
*A* Incident active TB incidence in PWH enrolled in the SHCS. Incidence per 1000 person y of incident TB, that is, at least 6 months after SHCS enrollment. *B*, Time from SHCS enrollment until incident TB. *C*, Prevalent active TB, active TB, that is, within 6 months after SHCS enrollment. Shown as prevalence among PWH newly enrolled in the SHCS. *D*, TB infection prevalence in the SHCS between 1988 and 2022 diagnosed by tuberculin skin test or interferon gamma release assay. Abbreviations: LTBI, latent TB infection; HIV, human immunodeficiency virus; PWH, people with HIV; SHCS, Swiss HIV cohort study; TB, tuberculosis.

Of 21 528 PWH, LTBI testing was performed in 14 684 (68.2%). Over time, between 40% and 60% were tested at SHCS enrollment, and a minority was tested post enrolment (Supplementary Figures 5 and 9). In total, 1233 (8.4%) PWH had a positive LTBI test: 338 (27.4%) IGRA and 498 (40.4%) TST test results were available before 6 months after SHCS inclusion; 145 (11.8%) IGRA and 252 (20.4%) TST results were available 6 months after SHCS inclusion. LTBI prevalence decreased from a peak of 15.1% in 2001% to 4.6% in 2021 (analysis 2.1 (Figure 1*B*), Figure 2*D*).

TB was diagnosed in 825 of 21 528 PWH. Of them, 277 (33.6%) were categorized as incident TB. Overall, TB cases decreased during the observation period: prevalent TB from a peak of 4.3% in 1993% to 0% in 2020 and incident TB from 90.8 cases per 1000 person-years from a peak in 1989 to 0.1 in 2021 (analysis 1 [Figure 1*A*]; Figure 2*A*, 2*C*). The median time from SHCS entry to incident TB was 3.2 (interquartile range [IQR] 1.5, 6) years (analysis 2.4; Figure 1*B*, Figure 2*B*).

### LTBI Testing Sensitivity and Preventive Treatment Effectiveness/Efficacy

**Figure 3.**
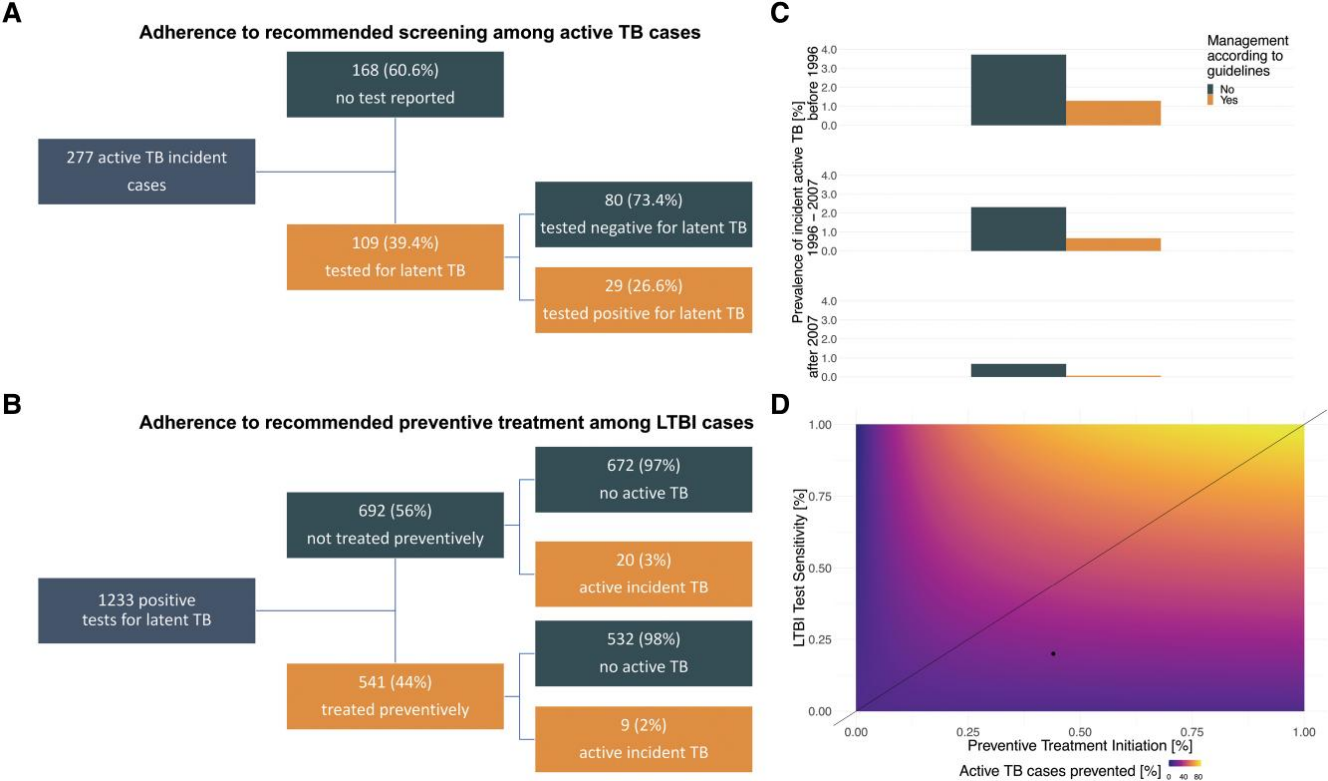
Adherence to TB management in PWH with latent (LTBI) or active TB in the SHCS. *A*, PWH with incident active TB stratified by their prior received LTBI test and its result. *B*, PWH with a positive or borderline LTBI test stratified by received preventive treatment and occurrence of incident active TB. *C*, Proportion of incident active TB based on adherence to LTBI test and treatment strategies. Stratified by SHCS enrollment before 1996, between 1996 and 2007, and after 2007. *D*, Numerical solution of a statistical model, comparing improvements in sensitivity of LTBI testing compared to adherence to preventive treatment initiation in their ability to prevent cases of incident active TB. Above the line sensitivity is higher, whereas below, initiation is higher. The dot indicates overall performance in the SHCS. Abbreviations: HIV, human immunodeficiency virus; LTBI, latent TB infection; PWH, people with HIV; SHCS, Swiss HIV cohort study; TB, tuberculosis.

Of 1233 PWH with a positive LTBI test, 541 (44%) initiated preventive treatment (Figure 3*B*). The median overall follow-up post positive LTBI test was 14 years (IQR 9, 21) (Supplementary Figure 3). After 16 years, 9 treated individuals and 20 non-treated individuals developed incident TB. The cumulative active TB incidence over 16 years was 2.3% (95% confidence interval [CI] .7%, 3.9%) in the treated and 3.2% (95% CI 1.8%, 4.6%) in the non-treated PWH. The absolute risk reduction is 0.9% (95% CI, −1.2%, 2.9%), the relative risk reduction 28.1% and the number needed to treat (NNT) prevent 1 incident TB case is 118 (95% CI 35, infinite). For NNT with the efficacy of preventive treatment, we considered 1 low ART adherent patient as non-treated and 1 patient with high frequency of traveling to the tropics (8 times, across 9 years, post treatment and pre-active TB) as reinfected. With the efficacy definition, the NNT is 53 (95% CI 26, infinite) (Supplementary Figures 1 and 8, also for NNTs at each survival time between 1 and 16 years). Sensitivity and specificity of LTBI testing for prediction of incident TB was 20% (95% CI 13%, 29%) and 95% (95% CI 95%, 95%), respectively.

For the overall adherence effectiveness to the TB specific prevention (analysis 2.3. [Figure 1*B*]), we split 20 980 PWH in 3 different time strata: SHCS enrolment time before 1996 (adherence 51% [95% CI 50%, 52%]), between 1996 and 2007 (adherence 77% [95% CI 76%, 78%]), and after 2007 (adherence 75% [95% CI 74%, 76%]). The proportion of incident TB was higher across strata in the PWH group not undergoing TB specific prevention adherence (before 1996, *P* < .0001; between 1996 and 2007, *P* < .0001; after 2007, *P* < .0001 [Figure 3*C*]). Overall, when adherent, the relative risk reduction was 76% (95% CI 69%, 83%) and the absolute risk reduction −2.1% (95% CI −2.5%, −1.7%).

Among the 277 PWH with incident active TB, 168 (60.1%) did not have a prior reported LTBI test. Of the remaining 109 LTBI tested, 80 (73.4%) had negative tests prior to incident TB (Figure 3*A*).

**Figure 4.**
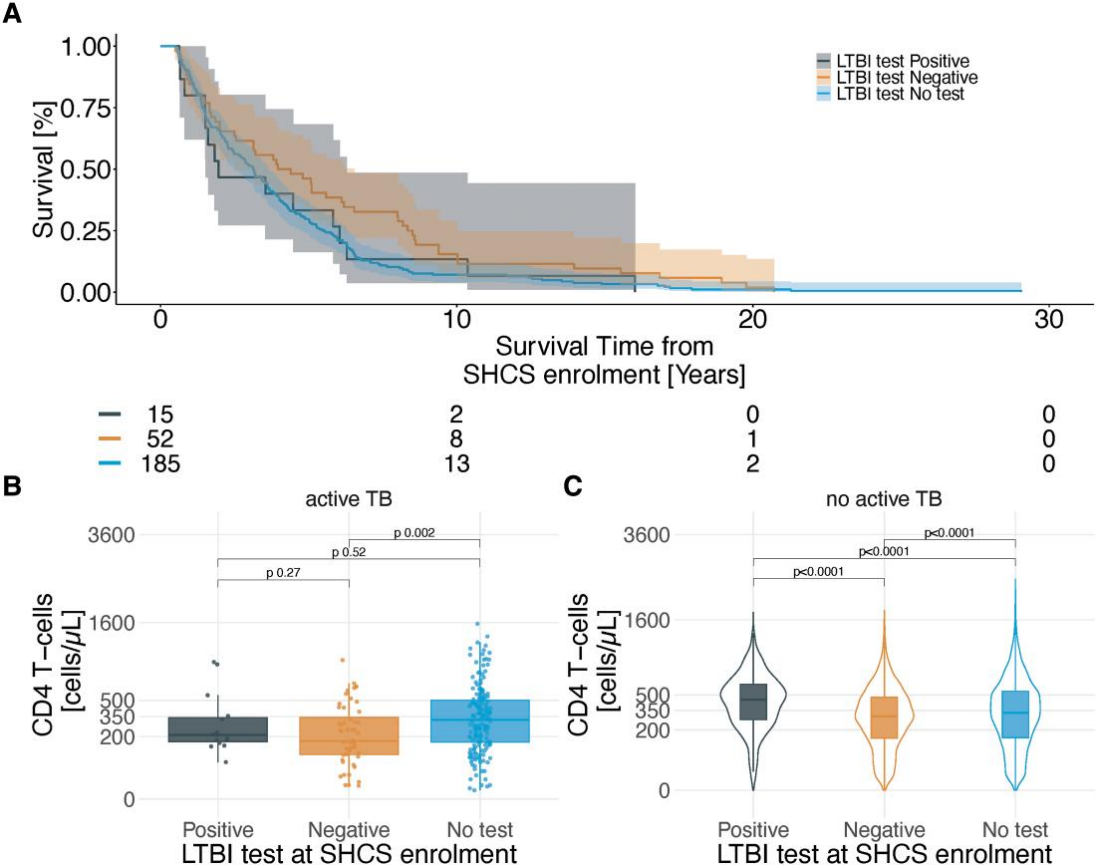
Comparison of CD4 T-cell count and LTBI test results/availability and the time to occurrence of incident active TB in people with HIV. LTBI test was at enrollment into the SHCS. TB is defined as TB at least 6 months after SHCS enrollment. LTBI test is defined as a positive/borderline or negative TST or IGRA or no available test within six months of SHCS enrolment. CD4 T-cell count is defined as the nadir measurement within six months of SHCS enrolment. *A*, Kaplan-Meier plot comparing the time until incident active TB, stratified by LTBI test results/availability. *B*, CD4 T-cell count stratified by LTBI test result/availability. *C*, CD4 T-cell count stratified by LTBI test result/availability among PWH without subsequent incident TB. Abbreviations: HIV, human immunodeficiency virus; LTBI, latent TB infection; PWH, people with HIV; SHCS, Swiss HIV cohort study; TB, tuberculosis; TST, tuberculin skin test; IGRA, interferon gamma release assay.

CD4 T-cell count at SHCS enrolment in PWH with subsequent TB was higher in LTBI non-tested compared to negative tested (analysis 5.2. [Figure 1*E*], *P* .002) (Figure 4*B*). Time to development of incident TB (analysis 5.1. [Figure 1*E*]) was shorter in non-tested compared to negative tested (HR 1.5, 95% CI 1.11, 2.1, Figure 4*A*). In PWH without subsequent TB, CD4 T-cell count was highest in LTBI positive tested, followed by non-tested (compared to positive *P* < .0001), and negative tested (compared to positive *P* < .0001) (Figure 4*C*).

### Counterfactual Improvements of Latent TB Test Sensitivity and Preventive Treatment Initiation

To quantify the effects improved LTBI testing sensitivity and treatment initiation may have, we performed counterfactual modelling (analysis 8 [Figure 1*H*]). We varied LTBI test sensitivity and treatment initiation proportion when LTBI test was positive. In the scenarios with higher sensitivity than treatment initiation (as indicated by the 45° line in Figure 3*D*), on average 45.5% of incident TB cases are prevented, whereas only 35.9% are prevented with higher treatment initiation (Difference: 9.6%, 9.5%, 9.7%).

### Risk Factors for Incident Active TB

After exclusion of PWH with prevalent TB, missing LTBI test, or missing risk factors, 12 783 PWH (73 incident TB cases) remained for the risk factor analysis (analysis 6 [Figure 1*F*], Table 1).

**Figure 5.**
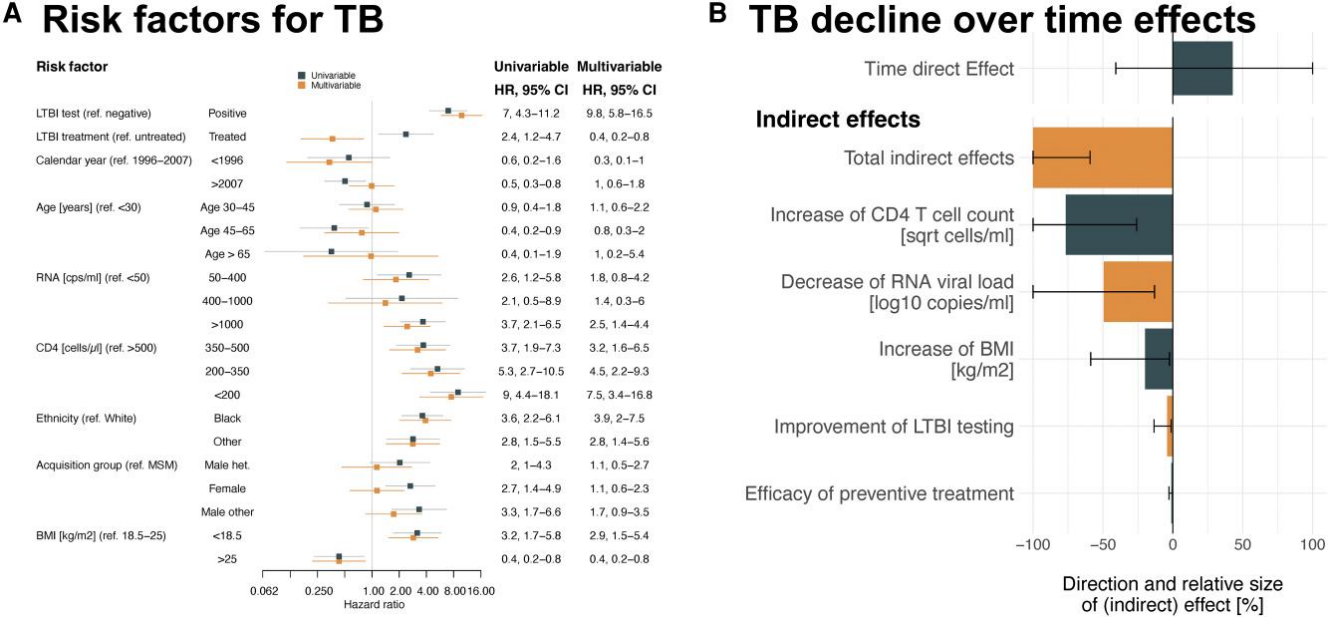
*A*. The hazard ratios for acquiring incident active TB, in PWH in the SHCS, using time updated uni-/multivariable Cox proportional hazard regression. *B*, Multiple mediation analysis, showing how increase of CD4 T cells, decrease of HIV-1 RNA viral load, preventive treatment efficacy, and LTBI testing improvements mediate the effect of time on incident active TB. All confidence intervals are clipped at ± 100%. Abbreviations: BMI, body mass index; CI, confidence interval; HIV-1, human immunodeficiency virus type 1; HR, hazard ratios; LTBI, latent TB infection; PWH, people with HIV; SHCS, Swiss HIV cohort study; TB, tuberculosis.

Several epidemiological and virological factors were associated with incident TB (Figure 5*A*, Supplementary Figure 4). Preventive treatment showed a protective effect against incident TB (HR 0.4, 95% CI .2, .8) (Figure 5*A*) (with preventive treatment efficacy definition, HR 0.2, 95% CI .1, .7, Supplementary Figure 2).

To only include PWH in times of potent ART availability, we excluded measurements before 1996 for the mediation analysis, after which 12 457 PWH remain (66 with incident TB) (analysis 7 [Figure 1*G*], Table 1). The incident TB decline, comparing the period 1996–2007 to 2007–2021, is a combination of natural indirect effects dominated by CD4 T-cell increase (relative indirect effect −77%, 95% CI −100%, −26%) and plasma RNA viral load suppression (relative indirect effect −49%, 95% CI −100%, −13%) (Figure 5*B*). This is in line with the median time until ART initiation from estimated time since infection, which is decreasing over the observation time (Supplementary Figure 11), while median CD4 T-cell count at ART initiation is increasing over observation time (Supplementary Figure 10). Conversely, preventive treatment completion is decreasing after peaking in 2014 (Supplementary Figure 12).

## DISCUSSION

Prevention of incident TB is part of good clinical practice in PWH [22]. Typically, PWH undergo risk stratification followed by preventive treatment. Most clinicians rely on three main criteria: a net immunodeficiency estimate (eg CD4 T-cell count), MTB specific T cell responses (“LTBI test”), and active TB exclusion [22]. It is well established that LTBI test performance is affected by HIV [23, 24]. However, lacking better alternatives, many clinicians and guidelines rely on TB specific prevention as outlined in guidelines across the world [25, 26].

Our evaluation of the TB specific prevention shows fewer incident TB cases in PWH treated according to guidelines on a patient level. We confirmed the protective effect of preventive treatment in our multivariable model, especially when we used its efficacy definition (ie, PWH with high treatment adherence) as in a previous analysis [17].

On a population level, however, our analysis demonstrated significantly reduced TB specific prevention effectiveness. Many factors contribute to this finding, including suboptimal performance of immune-based tests in PWH, ineffective preventive treatment schemes, poor adherence of both PWH to prescribed treatment and of physicians to recommend TB specific prevention [26]. This illustrates the widely recognized difference between efficacy and effectiveness of an intervention [27].

Physicians should follow the notion “intention to test is intention to treat” [28]. The observation that 56% of PWH with a positive LTBI did not receive preventive treatment reflects surprisingly poor acceptance of TB specific prevention. Independently of the acceptance of the recommendation, it may be difficult to convince a patient to start antiretroviral therapy, potentially treatment or prophylaxis for other opportunistic infection(s), and preventive therapy for MTB infection. These difficulties notwithstanding, once the patient is HIV-1 suppressed, preventive therapy can and should be offered for PWH with a positive LTBI test.

Our analysis demonstrates that the PWH at risk identification remains challenging; 73.4% of all tested PWH who developed TB during the follow-up were negative in the LTBI screening test. Our model suggests that even subtle screening sensitivity improvements can lead to substantial TB reduction.

As we investigate the TB prevention effectiveness of the entire approach, limitations accumulate at every step.

A potential bias for the poor performance of TB specific prevention are infections with MTB after testing or preventive treatment. Especially, tropical travelling as proxy for reinfection and low ART adherence as proxy for low preventive treatment adherence might be unreliable. However, infection/transmission within Switzerland is rare because Switzerland is a low endemic country [18]. Although certain sub-populations (eg, IVDU, shelters for unhoused persons, prisons, or high-density housing alongside persons immigrating from high TB endemic settings) may have undetected transmission in local clusters and be therefore at higher risk (eg, transmission cluster in Bern in homeless individuals and substance abusers) [29]. PWH may be at higher risk based on higher representation in these sub-populations despite living in a low endemicity setting. We corrected for age, ethnicity, and HIV acquisition mode but cannot exclude remaining selection bias. Overall, it is very likely that we missed some transmitted active TB cases. However, the overall trend is concordant with the data on a national level [30].

For PWH born abroad, it is possible they got infected while visiting friends and relatives, although this is improbable for the majority of LTBI negative PWH, because there is no difference between diagnosis of TB in positive and negatively tested PWH relative to LTBI test date. If negative PWH were infected later, they should on average develop TB later relative to LTBI test date compared to those PWH who tested positive.

A different explanation for the poor test performance could be due to differences in CD4 T-cell count. However, CD4 T-cell counts of PWH who developed TB were not different between PWH tested positive and PWH tested negative for LTBI. This observation suggests that the intuitive correlation between positive LTBI test and absolute numbers of circulating CD4 T-cells may not hold true for PWH who develop incident TB in the future. In contrast, PWH who tested LTBI positive but did not develop TB showed the expected correlation between positive LTBI testing and CD4 T-cell count. These observations suggests that poor sensitivity is not only a quantitative but also qualitative problem of circulating T-cells.

Finally, prophylaxis and treatment of opportunistic infections, (for example *Pneumocystis jiroveci* prophylaxis), swift correction of nutritional deficiencies and treatment of unrelated illnesses contribute to an overall improvement of the immune system [31]. This bundle of interventions thins out the effect of a single intervention, such as preventive treatment.

Additional limitations are the lack of direct adherence information in preventively treated PWH and missing data for the time period with high TB incidence (before 1996). Shorter therapy schedules for preventive treatment will likely improve adherence but were introduced too recently to affect the current investigation.

Our findings have two main implications: First, early HIV treatment is crucial. Suppressing HIV viremia and restoration of the immune system were in our study the strongest factors for TB reduction on a population level. Once HIV viremia is suppressed, preventive treatment should be offered to PWH with a positive LTBI test.

Second, negative LTBI testing does not rule out a high TB risk. PWH who are at high risk (eg, recent exposure) and have a negative LTBI test should still be regarded as high risk and preventive treatment should be considered, independently of CD4 count.

In summary, incident TB has been substantially declining in PWH in Switzerland. The main driver of this progress is successful HIV treatment. While reducing risk in positively tested PWH, TB specific prevention misses more than half of all PWH who develop TB. Consequently, the identification of PWH at highest risk for incident TB needs to be improved.

## Supplementary Data

Supplementary materials are available at *Clinical Infectious Diseases* online. Consisting of data provided by the authors to benefit the reader, the posted materials are not copyedited and are the sole responsibility of the authors, so questions or comments should be addressed to the corresponding author.

## Supplementary Material

ciad330_Supplementary_Data

## Notes

***Author Contributions*.** Conceptualization: M. Z., B. T., K. K., R. D. K., and J. N. Data curation: M. Z., B. T., K. K., C. R., M. K., J. No., H. F., M. H., H. H. H., A. C., M. C., N. D. L., E. B., D. L. B., H. F. G., R. D. K., and J. N. Formal analysis: M. Z. Funding acquisition: M. Z., R. D. K., and J. N. Investigation: M. Z., R. D. K., and J. N. Methodology: M. Z., R. D. K., and J. N. Project administration: R. D. K. and J. N. Resources: K. K., C. R., M. K., J. No., H .F., M. H., H. H. H., A. C., M. C., N. D. L., E. B., D. L. B., H. F. G., R. D. K., and J. N. Supervision: H. F. G., R. D. K., and J. N. Validation: R. D. K. Visualization: M. Z. and L. B. Writing—original draft: M. Z., K. K., L. B., R. D. K., and J. N. Writing—review and editing: M. Z., B. T., K. K., C. R., M. K., L. B., J. No., H. F., M. H., H. H. H., A. C., M. C., N. D. L., E. B., D. L. B., H. F. G., R. D. K., and J. N.

***Acknowledgments*.** The data are gathered by the Five Swiss University Hospitals, 2 Cantonal Hospitals, 15 affiliated hospitals, and 36 private physicians (listed in http://www.shcs.ch/180-health-care-providers). The authors thank the patients who participated in the Swiss HIV Cohort Study; the physicians and study nurses for the excellent patient care provided to participants; Jan Meier, Yves Schäfer, and Océane Follonier from the Swiss HIV Cohort Study data center for data management; and Danièle Perraudin and Marianne Amstad for administration.

***Financial support***. This work was supported by the Swiss National Science Foundation (SNSF) in the framework of the Swiss HIV Cohort Study (grant numbers 201369), the Swiss HIV Cohort Study (project P 881), and the Swiss HIV Research Foundation. R.D.K. and J.N. were supported by the SNSF (grant 324730_207957 to R.D.K. and 310030_200407 to J.N.).

***Data sharing statement*.** The individual level data sets generated or analyzed during the current study do not fulfill the requirements for open data access:

1. The SHCS informed consent states that sharing data outside the SHCS network is only permitted for specific studies on HIV infection and its complications, and to researchers who have signed an agreement detailing the use of the data and biological samples; and
2. The data are too dense and comprehensive to preserve patient privacy in persons living with HIV.

According to the Swiss law, data cannot be shared if data subjects have not agreed, or data are too sensitive to share. Investigators with a request for selected data should send a proposal to the respective SHCS address (www.shcs.ch/contact). The provision of data will be considered by the Scientific Board of the SHCS and the study team and is subject to Swiss legal and ethical regulations and is outlined in a material and data transfer agreement.
