# Supplementary material for: Understanding the Decline of Incident, Active Tuberculosis in People With Human Immunodeficiency Virus in Switzerland
Source: Clin Infect Dis. 2023 May 31;77(9):1303–11. doi: 10.1093/cid/ciad330 (PMC10640694; doi:10.1093/cid/ciad330)
Supplement: ciad330_Supplementary_Data [file ciad330_supplementary_data.docx]

**Supplementary materials**

Supplementary figure 1 Number of people with HIV needed to treat (NNT) to prevent one case of incident tuberculosis (TB) within the specified survival times. Incident (TB) is defined as TB at least six months after enrolment into the Swiss HIV Cohort Study. Analysis is done for the effectiveness- and the efficacy definition of preventive treatment. Treatment according to effectiveness is defined as receiving preventive treatment in the form of rifampicin, rifabutin, pyrazinamide, or isoniazid. Treatment according to efficacy definition is defined as receiving preventive treatment in the form of rifampicin, rifapentine, rifabutin, pyrazinamide, or isoniazid and adhering to full recommended treatment duration. Further, for efficacy only, PWH with low ART adherence or traveling to tropical regions after treatment were counted as untreated. NNT is calculated using the Kaplan-Meier approach with different specified survival times and NNT values were censored at 1000.

Supplementary figure 2 Sensitivity analysis: efficacy of preventive treatment. The hazard ratios for acquiring incident tuberculosis (TB), in people with HIV in the Swiss HIV cohort study (SHCS), using time updated uni-/multivariable cox proportional hazard regression. The outcome, incident tuberculosis, is defined as TB at least six months after SHCS enrolment. Time zero is defined as the date of first latent TB (LTBI) testing. Individuals without the outcome were censored six months before their last SHCS follow-up. Risk factors definitions: Test is defined as the comparison of a negative vs a positive/borderline LTBI test (tuberculin skin test or interferon gamma release assay) at time zero. Treatment is defined as receiving preventive treatment in the form of rifampicin, rifapentine, rifabutin, pyrazinamide, or isoniazid and adhering to full recommended treatment duration. Full treatment duration was (in months) defined as isoniazid ≥6, rifampicin ≥4, rifabutin ≥4, rifapentine ≥4, pyrazinamide ≥9, or isoniazid plus rifampicin ≥3. In addition, PWH with low ART adherence or traveling to tropical regions after treatment were counted as untreated. RNA is measured in HIV-1 RNA copies/ml. CD4 T-cell count is measured in CD4 T-cells/µl. BMI is calculated as kg/m^2^.

Supplementary figure 3 Sensitivity analysis: time from LTBI positive test to active tuberculosis or end of follow-up in people with HIV (PWH) enrolled in the Swiss HIV cohort study (SHCS). Median time is 14 years, indicated by the black line. Positive LTBI test was defined as positive or borderline test result by tuberculin skin test or interferon gamma release assay. Documented LTBI tests before SHCS enrolment are included.

**Supplementary figure 4 Sensitivity analysis: region of origin.** The hazard ratios for acquiring incident active tuberculosis (TB), in people with HIV in the Swiss HIV cohort study (SHCS), using time updated uni-/multivariable cox proportional hazard regression. The outcome, incident active tuberculosis, is defined as active TB at least six months after SHCS enrolment. Time zero is defined as the date of first latent TB (LTBI) testing. Individuals without the outcome were censored six months before their last SHCS follow-up. Risk factors definitions: Test is defined as the comparison of a negative vs a positive/borderline LTBI test (tuberculin skin test or interferon gamma release assay) at time zero. Treatment is defined as receiving preventive treatment in the form of rifampicin, rifapentine, rifabutin, pyrazinamide or isoniazid. RNA is measured in HIV-1 RNA copies/ml. CD4 T-cell count is measured in CD4 T-cells/µl. BMI is calculated as kg/m^2^.

Supplementary figure 5 Latent tuberculosis (LTBI) testing over time between 1988 and 01.08.2022 among people with HIV (PWH) in the Swiss HIV Cohort Study (SHCS). A: Stratified by test types, i.e., TST (tuberculin skin test), IGRA (interferon gamma release assay), or unknown. B: Stratified by IGRA test type, i.e., Quantiferon in tube, Quantiferon liquid, TB spot, or other. C: Stratified by test result, i.e., positive or borderline and negative. D: LTBI testing frequency within newly enrolled PWH.

Supplementary figure 6 Sensitivity analysis: Travel history. The hazard ratios for acquiring incident active tuberculosis (TB), in people with HIV in the Swiss HIV cohort study (SHCS), using time updated uni-/multivariable cox proportional hazard regression. The outcome, incident active tuberculosis, is defined as active TB at least six months after SHCS enrolment. Time zero is defined as the date of first latent TB (LTBI) testing. Individuals without the outcome were censored six months before their last SHCS follow-up. Risk factors definitions: Test is defined as the comparison of a negative vs a positive/borderline LTBI test (tuberculin skin test or interferon gamma release assay) at time zero. Treatment is defined as receiving preventive treatment in the form of rifampicin, rifapentine, rifabutin, pyrazinamide or isoniazid. RNA is measured in HIV-1 RNA copies/ml. CD4 T-cell count is measured in CD4 T-cells/µl. BMI is calculated as kg/m^2^. Travel history is defined as visiting a tropical country within the last 6 month.

Supplementary figure 7 Visualization of the system of ordinary differential equations to determine the effect of preventive latent tuberculosis (LTBI) treatment initiation compared to improvements in LTBI diagnostic tools.
Abbreviations: s = Sensitivity, ts = Treatment-success, tipos = Treatment initiation proportion when LTBI test positive, tineg = Treatment initiation proportion when LTBI test negative, atb = Incidence of active TB.

Supplementary figure 8 Tuberculosis (TB) management in people with HIV (PWH) with a positive latent TB (LTBI) test in the Swiss HIV cohort study (SHCS). Stratified by preventive treatment and subsequent incident active TB. LTBI is defined as positive tuberculin skin test or interferon gamma release assay. Incident active TB is defined as active TB at least 6 months after SHCS enrolment. Preventive treatment is defined as antibiotic treatment with rifampicin, rifapentine, rifabutin, pyrazinamide, or isoniazid with full adherence to the recommended regimen and no possibility of reinfection (i.e., subsequent heavy travelling to tropical regions). Full treatment duration was (in months) defined as isoniazid ≥6, rifampicin ≥4, rifabutin ≥4, rifapentine ≥4, pyrazinamide ≥9, or isoniazid plus rifampicin ≥3.

Supplementary figure 9 Latent tuberculosis (LTBI) testing frequency among people with HIV (PWH) in the Swiss HIV Cohort Study (SHCS). Frequency of LTBI test per year beyond 180 days post SHCS enrolment. LTBI tests are either a tuberculin skin test or an interferon gamma release assay.

Supplementary figure 10 Median CD4 T cell count at initiation of anti-retroviral therapy among people with HIV (PWH) in the Swiss HIV Cohort Study (SHCS).

Supplementary figure 11 Median time until initiation of anti-retroviral therapy (ART) among people with HIV (PWH) in the Swiss HIV Cohort Study (SHCS). Time is calculated between estimated infection date and first reported ART intake.

Supplementary figure 12 Tuberculosis preventive therapy (TPT) completion rate among people with HIV (PWH) in the Swiss HIV Cohort Study (SHCS).
